# GM-CSF-mediated inducement of bone marrow MDSCs by TSA and effect on survival of graft in mice

**DOI:** 10.1186/s40001-022-00788-8

**Published:** 2022-08-29

**Authors:** Shuguang Zhao, Shaohua Li, Jingci Yang, Weinian Gao, Ziying Chen

**Affiliations:** 1 Department of Cardiac Surgery, The Second Hospital of Hebei Medical University, 215 Heping West Road, Shijiazhuang, 050000 China; 2 Department of Respiratory Medicine, The First Hospital of Hebei Medical University, Shijiazhuang, China

**Keywords:** Myeloid-derived suppressor cells, GM-CSF, HDAC inhibitor, Differentiation, Transplant

## Abstract

**Objective:**

This study analyzed the effect of HDAC inhibitor, trichostatin A (TSA), in inducing granulocyte–macrophage colony-stimulating factor (GM-CSF)-mediated bone marrow (BM) cell differentiation to myeloid-derived suppressor cells (MDSCs) in vitro and in vivo.

**Methods:**

BM cell differentiation to CD11b + GR-1 + MDSCs was achieved by in vitro culture with TSA and GM-CSF, and the collected cells were analyzed by mixed lymphocyte culture to identify suppressive actions against effector T cells. RT-PCR and ELISA were conducted to analyze the CCL5 mRNA and protein levels in TSA + GM-CSF + BM, GR-1 + MDSCs and GR-1 + MDSC + CCL5 groups. The survival of cardiac grafts was compared between groups.

**Results:**

TSA was beneficial for the GM-CSF-mediated BM differentiation to CD11b + GR-1 + MDSCs. Adoptive transfer of GR-1 + MDSCs was powerful in suppressing CD4 + CD25-T cell proliferation and the effect was mediated by iNOS and HO-1; it also increased CCL5 gradient concentration between grafts and plasma to recruit Treg to grafts and prolong the survival of the grafts. Survival analysis revealed that the survival of grafts after adoptive transfer of GR-1 + MDSCs could be prolonged.

**Conclusion:**

This study helps in further research on the application value of MDSCs in the field of transplant, and may provide a new thought for the cell therapy in inducing immune tolerance in organ transplant.

## Introduction

Myeloid-derived suppressor cells (MDSCs) are a type of molecules expanded upon tumor formation, inflammation and surgical injury [1–5]. They are natural myeloid precursor cells with poor immunogenicity and remarkable immunosuppressive activity [1]. It is recognized that MDSCs can suppress the response and proliferation of T cells in a variety of ways. For instance, MDSCs affect the natural immune system by inhibiting natural killer (NK) cells and dendritic cells (DC) [1, 2]. Additionally, they can also induce the generation of natural regulatory T cells (nTreg) and inducible regulatory T cells (iTreg) with the B7-H1 pathway, and concurrently have chemotactic activity to advance peripheral Treg toward the location of tumor [6]. Schlecker et al. proved that peritumoral monocytic MDSCs (M-MDSCs) exerted chemotactic effect via the CCR5 chemokine receptor on Treg [7]. In recent years, the role of MDSCs in the field of organ transplant has emerged. It is reported that MDSCs have protective effect for organ graft by inducing specific immune tolerance [8, 9]. Mechanically, the immune tolerance is mainly attributed to the direct suppression of alloresponse or the synergic effects between MDSCs and Treg or NK cells. Adoptive transfer of MDSCs induced in vitro or in vivo might be a potential strategy for inducing and maintaining transplant tolerance.

MDSCs differentiation can be induced by a variety of factors, including multiple inflammatory factors and transcription factors, which have shown a close relationship with the differentiation and development of MDSCs [10]. Several factors are involved in the inducement of MDSCs, such as granulocyte–macrophage colony-stimulating factor (GM-CSF), granulocyte colony-stimulating factor (G-CSF), IL-1β, IL-6, IFN-γ, IL-10, IL-13 and lipopolysaccharide (LPS) [10]. Various cytokines were used as potential therapeutic targets in the stimulation of MDSC. Gal-1 had a dual immunomodulatory effect on monocyte-derived dendritic cells [11]. Trichostatin A (TSA) is the natural antifungal metabolite of Streptomyces that has efficient suppressive activity toward T cell receptor 1, a transcription factor and histone deacetylase (HDAC) [12]. It is proven that TSA is capable of inhibiting the maturation and differentiation of DC and augmenting the capability of Treg [13, 14]. In vitro studies revealed that TSA can directly inhibit HDAC to promote histone H3 lysine acetylation, thereby up-regulating the transcription of transcription factor Foxp3 to confer on T cells immunosuppressive activities [15]. HDAC inhibitor has shown powerful capacity in inducing immunosuppression, and it is proven that targeting HDAC can effectively regulate the graft-versus-host responses in allogeneic bone marrow (BM) transplant [16, 17].

In this study, we analyzed the effect of TSA in inducing GM-CSF-mediated BM cell differentiation to MDSCs, and also monitored the survival of grafts in mice.

## Materials and methods

### Animals

B6 (H2Kb) male mice were used as donors and BALB/c (H2Kd) mice were taken as recipients. Each mouse was weighed 18–22 g, aged 8–10 weeks and provided by Charles River (Beijing, China). Animal feed and excipient were given after ^60^Co radiation treatment. The mice were allowed to take clean drinking water and housed in specific pathogen-free (SPF) laminar air flow room of Hebei Medical University. The animal experiment was performed with the approval of Animal Care and Ethical Committee.

### Cell culture and flow cytometry sorting

Cells were isolated from donor mouse femur by flushing bones with 1 mL phosphate-buffered saline (PBS) supplemented with 5 mM EDTA plus 1% fetal calf serum using a 21 G needle attached to a 1 mL syringe. Cell suspension was generated by triturating cells through the needle. The BM cell suspensions were transferred onto a 40-um nylon sieve filter by flushing with normal saline solution, filtered, ground, and then suspended by RPMI-1640 medium. The prepared cell suspension was then placed in an incubator with CO_2_ for 6 h of culture. Following that, GM-CSF (1000 u/mL)-supplemented medium was added, or TSA (1 nM) was additionally supplemented on day 1/3/5. Non-adherent cells were collected on day 7 for flow cytometric analysis.

Flow cytometry was performed to sort MDSCs [18]. Cell phenotypes were analyzed using APC-conjugated anti-CD11b mAb (#17–0116-41, eBioscience, San Diego, CA, USA; marked with red) and FITC-conjugated anti-Gr1 mAb (#11–5931-82, eBioscience, San Diego, CA, USA; marked with blue). Gr-1 + MDSCs were sorted by labeling with FITC-conjugated anti-Gr1 mAb and anti-FITC immunomagnetic beads for positive selection (#130–048-701, Miltenyi Biotec, Bergisch Gladbach, Germany).

Splenic peripheral blood mononuclear cells (PBNCs) were labeled by PE-conjugated anti-CD4 antibody (#12–0042-82, eBioscience, San Diego, CA, USA; marked with green) and CD4 + T cells were sorted out by magnetic beads (anti-PE magnetic beads, #130–048-801, Miltenyi Biotec, Bergisch Gladbach, Germany). Additionally, the obtained CD4 + T cells were further labeled by FITC-conjugated anti-CD25 antibody (#11–0257-42, eBioscience, San Diego, CA, USA; marked with blue) to sort CD4 + CD25-Treg cells (negative selection) and CD4 + CD25 + Treg cells (positive selection). Appropriately conjugated species- and isotype-matched IgG served as controls. Analysis of stained cells was performed with a FACSCanto flow cytometer (BD Biosciences). The data were analyzed using FlowJo software.

### Mixed lymphocyte culture (MLC)

MLC was performed to analyze the activity of allogeneic effector T cells. CD4 + CD25-T cells were co-cultured with TSA + GM-CSF + BM cells, GR-1 + MDSCs, and CD4 + CD25 + Treg, respectively. BALB/c CD4 + effector T cells (4 × 10^5^) were mixed with γ-irradiated DCs (1 × 10^4^) for 72 h, followed by addition of TSA + GM-CSF + BM cells (1 × 10^5^), GR-1 + MDSCs (1 × 10^5^) and Treg (1 × 10^5^), respectively. To confirm the roles of inducible nitric oxide synthase (iNOS) and hemeoxygenase-1 (HO-1) in the suppressive actions of MDSCs, iNOS inhibitor L-NMMA (0.5 mM; Sigma-Aldrich) and HO-1 inhibitor SnPP (0.15 mM; Enzo Life Sciences, Farmingdale, NY, USA) were electively added in the GR-1 + MDSCs mixture, and 3H-TdR (1 ul per well) was then added 16 h before culture termination.

### Western blot

Cell lysates of GM-CSF + BM cells and GR-1 + MDSCs (1 × 10^6^) were obtained. After electrophoresis and membrane transfer, antigen–antibody reactions were performed by adding anti-acetyl-histone H4 (Lys8) antibody (#2594, Cell Signaling Technology, Beverly, MA, USA), anti-HO-1 antibody (#ENZ-ABS663, Enzo Life Sciences, Farmingdale, NY, USA), anti-iNOS antibody (#ab3523, Abcam, Cambridge, MA, USA), and anti-β-actin antibody (#NB600-501, Novus Biologicals, Littleton, CO, USA). The specific procedure was as described in literature [20].

### Cardiac grafting and grouping

Mice were firstly anesthetized by intraperitoneal injection of 1% 200 mg/kg pentobarbital sodium, and then systemically heparinized via infra-hepatic inferior vena cava (IVC) injection of 1 u heparin. Under ZEISS15 microscope, the donor heart was transplanted to the abdominal cavity of the recipient by IVC–IVC and aorta–abdominal aorta anastomoses. After successful transplant, the recipients were divided into three groups: adoptive transfer of TSA + GM-CSF + BM cells (TSA + GM-CSF + BM), adoptive transfer of GR-1 + MDSCs (5 × 10^7^) (GR-1 + MDSCs), and adoptive transfer of GR-1 + MDSCs (5 × 10^7^) plus tail vein injection of CCL5 (14 mg/kg/d) on day 2/5/8 (GR-1 + MDSCs + rCCL5). BALB/c mice with cardiac allografting were used as control. BM cells and MDSCs were injected through the tail vein, and TSA was given by oral (100 mg/kg, three times weekly). Survival of the cardiac grafts was assessed by the abdominal cardiac fluctuation. On day 10 after transplantation, cardiac graft tissue and peripheral plasma from 3 mice of each group were extracted and analyzed.

### Real-time quantitative polymerase chain reaction (qRT-PCR)

RNA was extracted from sorted cells or snap-frozen cardiac graft tissues with TRIzol reagent (Invitrogen, USA). Reverse transcription was performed using 2 mg RNA with the Omniscript RT-PCR Kit (QIAGEN). qRT-PCR was run on the Applied Biosystems GenAmp 7900 system. TaqMan Gene Expression Assay (Applied Biosystems, Life Technologies) was performed to quantitatively analyze the mRNA levels of β-actin (Rn 00667869_m1), CCL5 (Rn00579590_m1), Foxp3 (Rn01525085_m1), and Ccr5 (Rn00588629_m1). β-actin was used an endogenous control gene to achieve expression standardization. 2^ΔΔCT^ method was applied to calculate the relative expression of target gene.

### CCL5 enzyme-linked immunosorbent assay (ELISA)

Freshly extracted cells were cultured in complete medium (RPMI-1640, 10% FBS, 2 mM L-Glutamine, 100 U/ml penicillin, 0.1 mg/ml, streptomycin, 2-ME) at 37 °C and 5% CO_2_. After 16 h, the supernatant was collected and frozen at −220 °C. Heparinized blood samples were used to collect plasma, which was also restored at −220 °C. Cardiac grafts after 10 days of transplantation were extracted and the volume was evaluated. Additionally, the grafts were homogenated with RIPA lysis on ULTRATURRAX (IKA, Lille, France) at 4 °C. After stirring, the lysates were centrifuged. The supernatant was collected and analyzed by CCL5 ELISA Kit (Proteintech, US) following the standard instructions.

### Statistical analysis

Results are expressed as means ± SD. Significant differences between means were determined using a one-tailed Student's t test, and P < 0.05 was considered significant. Graft survival data were calculated using the Kaplan–Meier method. The log-rank test was used to compare survival times between different groups. Graphs were generated using GraphPad Prism, v8.0 (GraphPad Software, La Jolla, CA, USA).

## Results

### TSA facilitates the GM-CSF-induced differentiation of BM cells to MDSCs

**Fig. 1:**
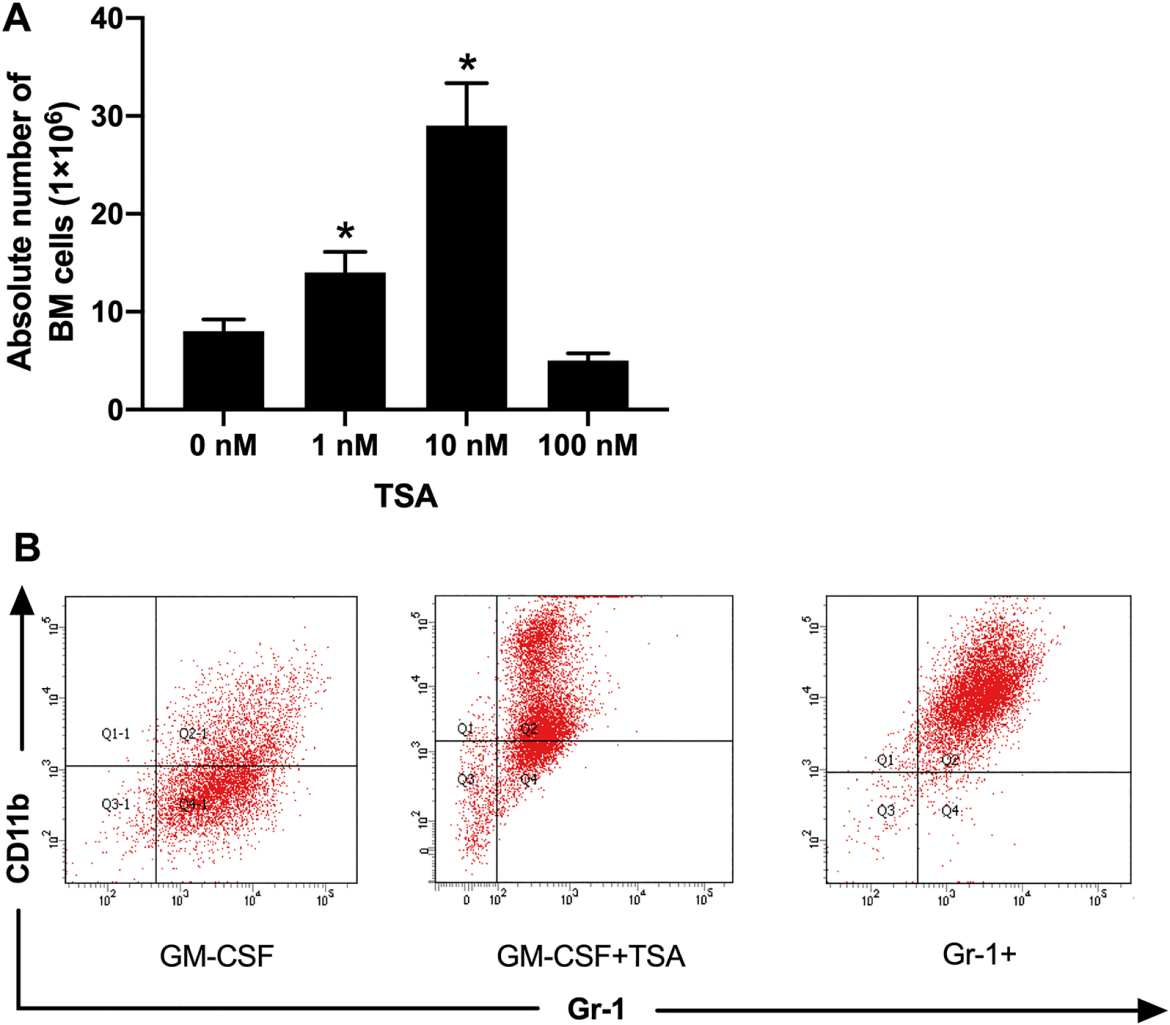
TSA can promote the GM-CSF-induced BM cell differentiation and increase the production of CD11b + Gr-1 + MDSCs. **A** TSA can promote the GM-CSF-induced BM cell differentiation. TSA was added to the culture medium containing 1000 u/ml GM-CSF to induce the differentiation of BM cells from B6 mice. Mean ± SD was obtained from 3 independent experimental results. * *P* < 0.05, compared to the 0 nM group; **B** TSA can promote the production of CD11b + Gr-1 + MDSCs. The three images, respectively, show the cells cultured with GM-CSF, GM-CSF + 10 nM TSA, and high-quality CD11b + Gr-1 + MDSCs sorted by flow cytometry. Cell phenotype was analyzed in flow cytometry. Each experiment was performed in triplicate

The effect of TSA on the GM-CSF-induced differentiation of BM cells was firstly analyzed. TSA was supplemented to the GM-CSF culture medium and was found to accelerate the proliferation of BM cells. Relative to the single use of GM-CSF, co-culture of TSA (1 nM) could slightly increase the proliferation of BM cells. Notably, the proliferation capability could be enhanced three times when TSA was applied at 10 nM, yet the cell proliferation was suppressed upon a higher concentration, which might be the result of cell apoptosis (Fig. 1A).

In the context of BM cell proliferation augmentation by GM-CSF, Gr-1 cell count was small. The number of CD11b + Gr-1 + cells was remarkably increased after an addition of 10 nM TSA. At this time, CD11b + Gr-1 + MDSCs of 98% purity could be obtained by magnetic bead sorting (Fig. 1B).

### The MDSCs induced by TSA + GM-CSF have immunosuppressive activity

**Fig. 2:**
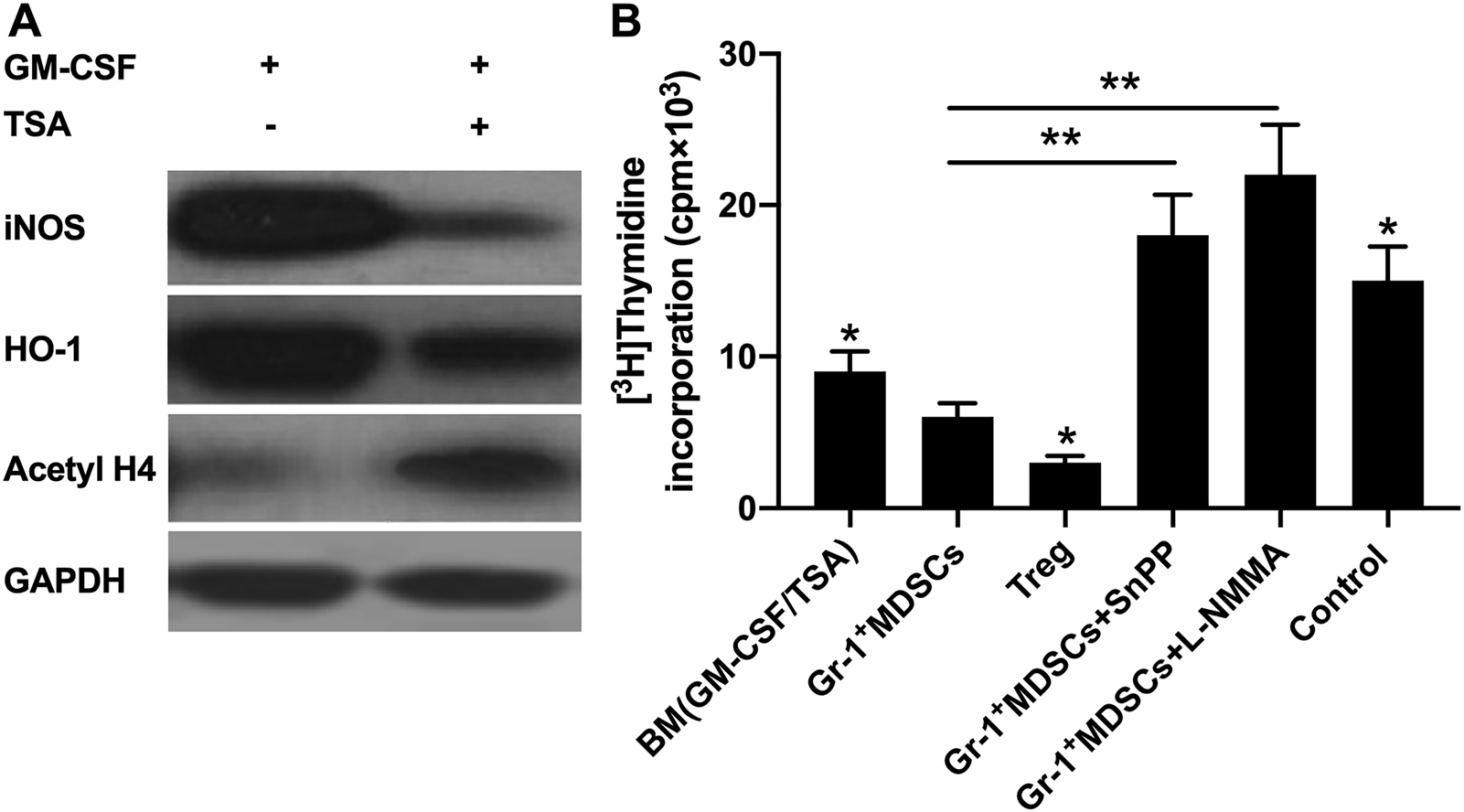
The MDSCs induced by TSA + GM-CSF has immunosuppressive activity. **A** Western blot showed decreasing iNOS and HO-1 expression while increasing histone H4 acetylation level in GM-CSF-BM cells after addition of TSA. GAPDH was the reference. MDSCs were obtained by magnetic bead sorting from cells cultured with GM-CSF or 10 nM TSA-GM-CSF after 7 days; **B** iNOS and HO-1 are indispensable in the suppressive actions of GM-CSF/TSA-GM-CSF stimulated MDSCs for T cell proliferation. The number of MDSCs was 3 × 10^4^. iNOS inhibitor L-NMMA and HO-1 inhibitor SnPP were added to culture medium from day 0. Each experiment was performed in triplicate

It is commonly believed that iNOS and HO-1 are key in the suppressive actions of MDSCs for T cells [1, 19]. Here, western blot was performed to find that, iNOS and HO-1 expression decreased in BM cells after addition of TSA versus the single treatment with GM-CSF, and the histone H4 acetylation level increased concomitantly (Fig. 2A).

In the three MLC groups (CD4 + CD25-T plus TSA + GM-CSF + BM, CD4 + CD25-T plus GR-1 + MDSCs, CD4 + CD25-T plus CD4 + CD25 + Treg), application of L-NMMA and SnPP suppressed the expression of iNOS and HO-1. We noted that the TSA + GM-CSF + BM cells had more potent suppressive effect on CD4 + CD25-T cell proliferation than GR-1 + MDSCs, while weaker versus CD4 + CD25 + Treg. The decreasing immunosuppressive activity of GR-1 + MDSCs indicated that the GR-1 + MDSCs cultured in TSA had lower expression levels of iNOS and HO-1. However, the role of iNOS and HO-1 enzymes involved in the action of MDSCs on T cells could not be negated (Fig. 2B).

### qRT-PCR identifies high expression of CCL5 and Foxp3 mRNA

MDSCs are important cells that secrete CCL5, by which immune response blockade can be achieved [20]. After successful heart transplant, the BALB/c recipients were divided into three groups: TSA + GM-CSF + BM, GR-1 + MDSCs, and GR-1 + MDSCs + CCL5. BALB/c mice with cardiac allografting were used as control.

**Fig. 3:**
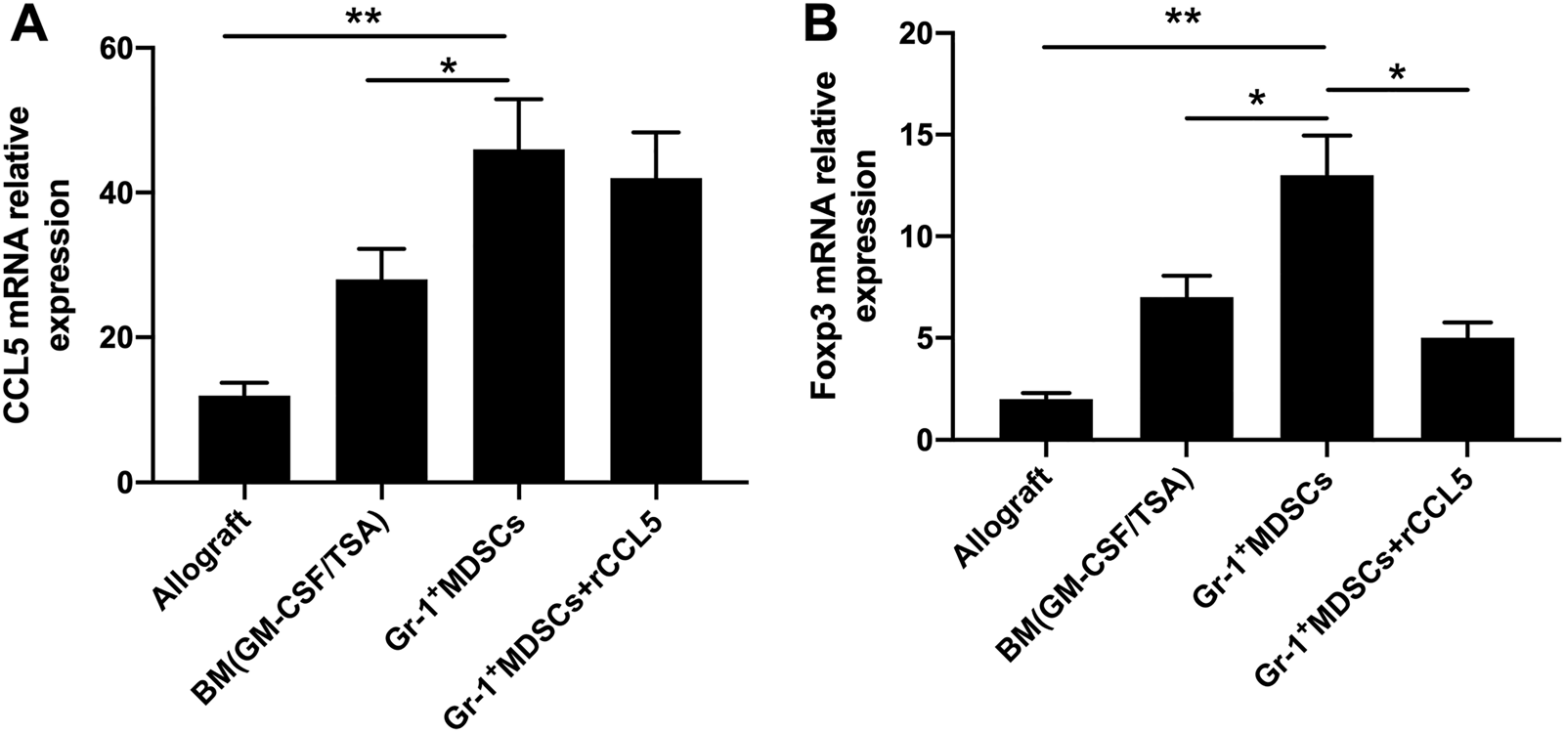
qRT-PCR identifies CCL5 and Foxp3 mRNA expression in cardiac grafts. **A** CCL5 mRNA expression (allograft, *n = *4; TSA + GM-CSF + BM, *n = *6; GR-1 + MDSCs, *n = *5; GR-1 + MDSCs + CCL5, *n = *4); **B** Foxp3 mRNA expression (allograft, *n = *3; TSA + GM-CSF + BM, *n = *6; GR-1 + MDSCs, *n = *5; GR-1 + MDSCs + CCL5, *n = *4). * *p* < 0.05, **p < 0.01. P value was calculated by Student's t test, and *p < *0.05 was considered as statistical significance. Each experiment was performed in triplicate

On day 10 of transplantation, RNA of cardiac grafts from each group was analyzed with qRT-PCR. It was found that the CCL5 mRNA of the grafts with adoptive transfer of GR-1 + MDSCs was the highest among the four groups. It was noting that the additional injection of CCL5 did not significantly change the CCL5 mRNA expression (Fig. 3A).

Foxp3 is a member of the forkhead transcription factor family and regarded as a marker of Treg. We found that the changing trend of the transcriptional level of Foxp3 was consistent with that of CCL5 in the four groups (Fig. 3B). The highest level was seen in the xenografts with adoptive transfer of GR-1 + MDSCs, and approximately 10 times larger than that in the allografts. Additionally, the injection of CCL5 decreased Foxp3 mRNA in xenografts. We believed that the grafting induced the generation of a local inflammatory environment, where a large number of inflammatory factors and chemokines were released, which might be beneficial for the colonization of GR-1 cells into the allografts.

### CCL5 gradient concentration between grafts and plasma and Treg aggregation in grafts

**Fig. 4:**
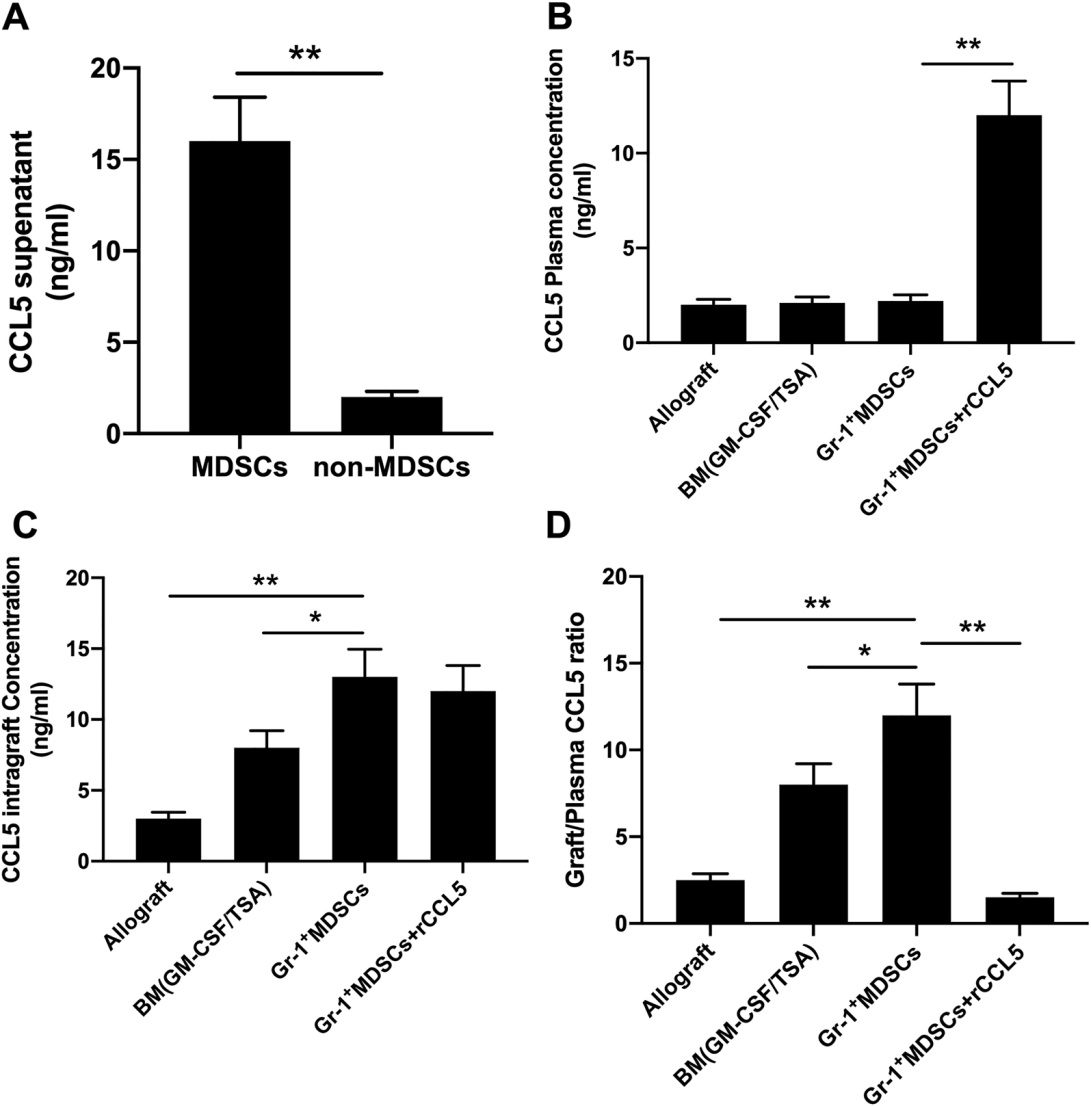
ELISA assay measures CCL5 protein content in MDSCs, grafts and peripheral plasma. **A** MDSCs from GR-1 + MDSCs (*n = *4), and non-MDSCs from GR-1-MDSCs (*n = *4); **B**–**D** CCL5 protein content in graft and plasma of each group, and corresponding graft/plasma ratio (allograft, *n = *3; TSA + GM-CSF + BM, *n = *6; GR-1 + MDSCs, *n = *5; GR-1 + MDSCs + CCL5, *n = *4). * *p < *0.05, ** *p < *0.01. Each experiment was performed in triplicate

Chemokines are significant participants in cell migration, and the gradient concentration is more beneficial for cell localization. In this context, gradient chemokine must exist during the migration of Treg into the grafts. ELISA assay was performed to identify that the MDSCs induced by TSA in vitro could secrete CCL5, as demonstrated by increasing CCL5 protein content in the cell culture supernatant (Fig. 4A).

On day 10 of transplantation, cardiac graft tissue and peripheral blood were sampled, and CCL5 proteins were further examined. It was noted that the difference among peripheral plasma of each group in CCL5 proteins was not remarkable (Fig. 4B). Of note, significant difference was observed between cardiac graft tissue and plasma, and the CCL5 content in xenografts was 50 times larger versus that in plasma. GR-1 + MDSCs group showed higher CCL5 level (Fig. 4C) and graft/plasma ratio (Fig. 4D) relative to the TSA + GM-CSF + BM group and the allografts. In the GR-1 + MDSCs + CCL5 group, CCL5 proteins were abundant in both plasma and grafts, whereas the graft/plasma ratio was the lowest among the four groups.

### Adoptive transfer of MDSCs can prolong the survival of cardiac graft

**Fig. 5:**
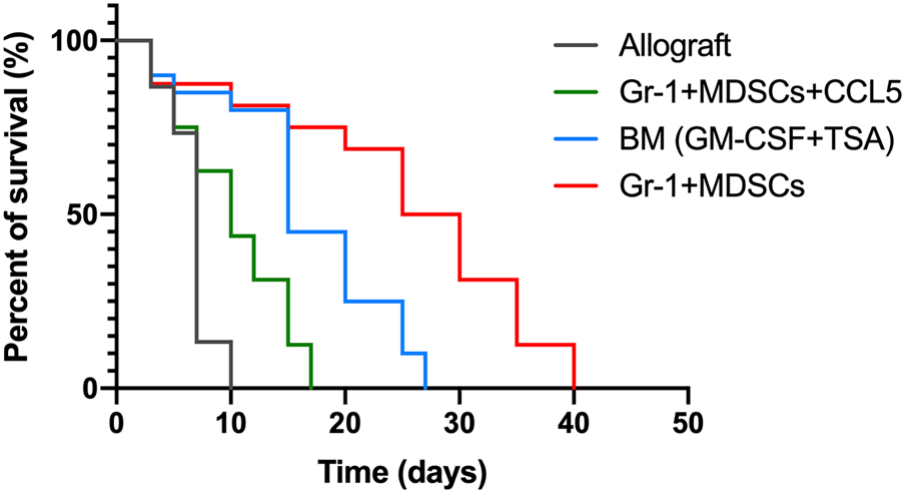
Survival of mice in each group (allograft, *n = *17; TSA + GM-CSF + BM, *n = *14; GR-1 + MDSCs, *n = *15; GR-1 + MDSCs + CCL5, *n = *16). The experiment was performed in triplicate

The time to complete cardiac arrest was defined as the rejection time. It was found that the syngeneic grafts could survive in the long term, while the allografts without cell therapy almost died within 10 days of transplant (Fig. 5). Additionally, the mice receiving MDSCs and CCL5 treatment had a 10-day survival rate of 30%, and all died within 20 days. Of the four groups, the GR-1 + MDSCs group had a higher survival rate than the TSA + GM-CSF + BM group, with a 20-day survival rate of 60%. However, all mice in the GR-1 + MDSCs group died within 40 days. This implied that GR-1 + MDSCs treatment could suppress acute immune rejection, yet it still failed to establish immune tolerance and maintain the long-term survival of the grafts. Moreover, it was also noted that the injection of CCL5 was not beneficial for the survival of grafts, but the survival time was still longer than that of the untreated grafts.

## Discussion

In the present study, high-dose GM-CSF and HDAC inhibitor TSA were combined to achieve BM cell differentiation to BM-MDSCs in vitro. Adoptive transfer was devised to prove that the BM-MDSCs were capable of recruiting Treg to grafts and prolonging the survival of the grafts. This provides a new thought for the inducement of immune tolerance in organ grafts by cell therapy.

We here found that TSA supplemented to GM-CSF culture medium promoted the proliferation of BM cells and increased GR-1 expression. In the immunoprecipitation experiment, iNOS and HO-1 expression decreased in GR-1 + cells after TSA application. We believed that TSA suppressed the transcription of iNOS and HO-1, and the low levels of iNOS and HO-1 are a basis for the immunosuppressive action of MDSCs, which can be deprived by L-NMMA (iNOS inhibitor) and SnPP (HO-1 inhibitor). Additionally, TSA could remarkably augment the level of histone H3 acetylation [12]. It was speculated that the acetylation of histone H3 might regulate the transcription factors closely associated with the generation of MDSCs, such as STAT3 and C/EBPβ, thereby affecting the expression of inflammation-related genes. MLC assay showed that the obtained GR-1 + cells had strong power to suppress CD4 + effector T cells, weaker than natural Treg. Hence, we believed that Treg remain the core players in the inducement of immune tolerance in grafts.

Schlecker et al. proved that peritumoral M-MDSCs exerted chemotactic effect via the CCR5 chemokine receptor on Treg [7]. In solid tumors, overexpressing CCL5 by tumor cells will lead to the recruitment of Treg and establishment of local immunosuppression. Similar condition might be seen in cardiac grafts and related to the localization of Treg as well as the establishment of immune tolerance. CCL5 commonly expresses in inflammatory environment and plays a part in tumor-associated immunoregulation [21, 22]. It is known that the CCL5 expression in allografts can also induce the recruitment of Treg [20]. Here, we found that the adoptive transfer of GR-1 + MDSCs resulted in increasing CCL5 mRNA in cardiac grafts, and the expression of Foxp3 exhibited the same expression changing trend. Following injection of CCL-5, the high CCL5 in peripheral plasma was found to bring no benefit for the recruitment of Treg to grafts, yet the Foxp3 expression in xenografts was still higher than the allografts without any cell therapy. There might be some chemokines involved.

Furthermore, the difference in CCL5 proteins in peripheral plasma and graft tissue was analyzed. It was found that the adoptive transfer of MDSCs did not change the plasma CCL5 level, and the GR-1 + culture supernatant had remarkably higher CCL5 protein level versus the GR-1− culture supernatant. The adoptive transfer of a large number of GR-1 + MDSCs increased the CCL5 protein level in grafts, which was considered a result of the CCL5 gradient concentration between the graft and peripheral environment due to the aggregation of GR-1 + MDSCs in grafts under the action of inflammatory factors. It is notable that the CCL5 graft/plasma ratio was the highest in the GR-1 + MDSCs group, more conducive to aggregation of peripheral Treg in grafts. It is a fact that the aggregation of MDSCs and Treg in grafts is beneficial for the establishment of local immunosuppressive environment and immune tolerance. Further survival analysis demonstrated that the mice with allografting and without any cell therapy survived in a short term, and the survival could be prolonged after adoptive transfer of GR-1 + MDSCs. It suggested that GR-1 + MDSCs cell therapy might suppress effector T cells directly or by coordination with Treg. However, it failed to establish immune tolerance and the consumption of MDSCs was incapable of maintaining the tolerance environment.

To conclude, this study obtained MDSCs of immunosuppressive activity by in vitro differentiation of BM cells with HDAC inhibitor, TSA, and achieved prolonged survival of xenografts in mice by adoptive transfer of MDSCs. The findings of the study may provide a new strategy for the cellular immunotherapy in grafts and help perform further in-depth research on the application value of MDSCs in the field of transplant.

## Data Availability

The data that support the findings of this study are available from the corresponding author upon reasonable request.
